# Stifle joint alterations in dogs with patellar luxation

**DOI:** 10.1038/s41598-026-44207-y

**Published:** 2026-04-02

**Authors:** Palkhi Sharma, Arun Anand, Devendra Pathak, Vandana Sangwan

**Affiliations:** 1 Department of Veterinary Surgery and Radiology, College of Veterinary Science, Guru Angad Dev Veterinary and Animal Sciences University, Ludhiana, Punjab 141004 India; 2 Department of Veterinary Anatomy, College of Veterinary Science, ORCID-iDs, Guru Angad Dev Veterinary and Animal Sciences University, Ludhiana, Punjab 141004 India

**Keywords:** Patellar luxation, Articular cartilage, Joint capsule, Canine, Surgery, Anatomy, Diseases, Medical research, Rheumatology

## Abstract

Patellar luxation results in abnormal positioning of the patella outside the trochlear groove, leading to microscopic and macroscopic alterations within the canine stifle joint. This study evaluated histopathological changes in the articular cartilage and joint capsule of dogs affected by patellar luxation, along with gross intra-operative findings observed during surgical correction. Articular cartilage and joint capsule samples from fourteen affected stifle joints and two control joints were examined histologically. Erosive lesions of articular cartilage were observed across multiple stifle joint subdivisions. Affected joints demonstrated inflammatory and degenerative changes in both articular cartilage and joint capsule. Quantitative analysis revealed significantly greater chondrocyte loss in Group III (Grade 4) cases compared with lower-grade luxation groups, while Safranin–O staining demonstrated significantly reduced proteoglycan content in Group III compared with control samples. Gross intra-operative assessment identified articular cartilage erosions, most frequently involving the distal patella and lateral trochlea, as well as variable degrees of osteophytosis. These findings indicate an association between patellar luxation and structural alterations of stifle joint tissues, with more pronounced changes observed in higher-grade cases.

## Introduction

One frequent orthopaedic condition that causes canine hindlimb lameness is patellar luxation^8^. According to reports, its incidence among canine orthopaedic problems ranges from 1.30 to 9.2%^2,9^. Small breed dogs are frequently found to have patellar luxation, with Chihuahuas, Poodles, Pomeranians, Maltese, and Bull terriers being overrepresented. The incidence of medial patellar luxation was greater in small-breed dogs^1^. Although patellar luxation is typically thought of as a developmental condition with a complicated aetiology, the two most prevalent aetiological patho-physiological mechanisms that cause it in dogs are congenital and traumatic^6^. The present study was envisaged with the hypothesis that the ectopic movement of the patella outside the trochlear groove leads to micro- and macroscopic changes in the stifle joint, observed histologically and grossly during corrective surgery.

## Materials and methods

This prospective study included 14 stifle joints from client-owned dogs diagnosed with grades 2, 3, or 4 of patellar luxation (PL), presented to the Teaching Veterinary Clinical Complex, Guru Angad Dev Veterinary & Animal Sciences University (GADVASU), Ludhiana, India, over a one-year period (March 2024–February 2025). Each stifle joint was considered as a single experimental unit.

### Ethics statement

All procedures were performed on client-owned dogs presented for clinical care at the Teaching Veterinary Clinical Complex, Guru Angad Dev Veterinary and Animal Sciences University, Ludhiana, India, with informed owner consent obtained prior to surgical management. The research protocol was reviewed and approved by the Institutional Animal Ethics Committee (IAEC) of Guru Angad Dev Veterinary and Animal Sciences University, Ludhiana, India, and subsequently approved by the Committee for the Control and Supervision of Experiments on Animals (CCSEA), Delhi (Approval number: GADVASU/2024/IAEC/71/05). All procedures were carried out in accordance with relevant guidelines and regulations.

### Inclusion and exclusion criteria

Dogs with confirmed grade 2–4 patellar luxation were included. Dogs with systemic conditions were excluded. No dogs meeting inclusion criteria were excluded from analysis.

Study groups

Dogs were divided into four groups according to the grade of luxation using Putnam’s grading system^10^, amended by Singleton^11^. The groups included:

- Group I: PL Grade 2 (*n* = 5).
- Group II: PL Grade 3 (*n* = 5).
- Group III: PL Grade 4 (*n* = 4).
- Control group: stifle joints from dogs without patellar luxation or other orthopedic abnormalities (*n* = 2).

### Experimental procedures

The clinical presentation and signalment of each dog were recorded. The study population included dogs of the following breeds: Pomeranian, Labrador Retriever, Golden Retriever, Cane Corso, Pug, and Shih Tzu.

Joint capsule and articular cartilage samples were collected during surgical arthrotomy performed for correction of patellar luxation.

The joint capsule samples were obtained by incising the joint capsule along the proximal-lateral edges of the incision made in the synovial membrane of the joint.

The articular cartilage samples were consistently harvested from the medial and lateral aspects of the femoral trochlear groove, corresponding to the primary patellofemoral contact regions commonly affected in patellar luxation. The sample was excised using a No. 10 Bard-Parker blade and was carefully elevated and separated from the underlying subchondral bone. Care was taken to preserve the tissue architecture during sampling, and no additional cartilage loss attributable to post-harvest handling was observed. Sampling was done from the same anatomical regions in all the cases to ensure comparability across groups. The samples for cases included in the control group (*n* = 2) were obtained post-euthanasia from dogs euthanised for non-orthopaedic reasons, with no evidence of stifle joint pathology on clinical examination.

Both the joint capsule and articular cartilage samples were fixed with 10% buffered formalin for a minimum of 72 h. The fixed tissue samples were processed routinely, embedded in paraffin, sectioned at 3–5 μm thickness, and stained with Haematoxylin and Eosin (H&E) for histological evaluation.

Histopathological evaluation was conducted by a histologist/pathologist to assess alterations in articular cartilage and joint capsule morphology, corresponding to any signs of patellar luxation or other stifle joint arthropathy. H&E-stained sections from dogs with patellar luxation were compared with samples obtained from control group.

For quantitative analysis, six random, non-overlapping microscopic fields were selected per articular cartilage section by systematically scanning the section at high magnification. Fields were chosen across the cartilage surface while avoiding areas with artefacts or tissue folding. The same field-selection approach was applied consistently across all samples to minimise observer bias. Chondrocyte counts were recorded for each microscopic field. Chondrocyte loss was operationally defined as the number of empty lacunae (lacunae lacking a visible nucleus) per microscopic field. Six microscopic fields were analysed per cartilage section.

Articular cartilage sample sections were further subjected to Safranin-O fast green staining to assess the cartilage’s glycosaminoglycan content, using a semi-quantitative histochemical scoring approach similar to that described by Wander et al.^12^. Staining intensity was evaluated in the superficial, intermediate and territorial zones of cartilage. Each zone was scored on basis of staining intensity of Safranin-O stain uptake on a four - point scale of 0 to 3 (0 = no stain uptake; 1 = 33% normal stain uptake; 2 = 66% normal stain uptake; 3 = normal stain uptake), and a total score out of 9 was assigned to each section. In the present study, regions of structurally intact cartilage within each section were used as internal comparison standards to guide relative staining intensity assessment, and scoring criteria were applied consistently across all samples.

The gross, macroscopic alterations visualised intra-operatively were recorded. The frequency of articular cartilage erosive lesions was recorded, and the distribution at six different pre-defined subdivisions of the stifle (Distal patella, medial patella, centre of patella, lateral trochlea, medial trochlea, and trochlear groove) was noted.

### Randomisation and blinding

Randomisation was not applicable, as all eligible clinical cases were included. Blinding was not performed, as the same clinical team conducted diagnosis, surgery and histopathological evaluation. This is acknowledged as a limitation of the study.

### Statistical analysis

Data were analysed using Microsoft Excel 2007 and GraphPad Prism 6 (PRISM^®^ ver. 6.01, GraphPad Software, Inc.). Descriptive statistics are presented as mean ± standard deviation (SD), with 95% confidence intervals where applicable. The primary outcome measure was Chondrocyte loss in articular cartilage. Normality of the chondrocyte loss data was assessed using the Shapiro–Wilk, Anderson–Darling, and Kolmogorov–Smirnov tests and was found to deviate from a normal distribution; therefore, group comparisons for chondrocyte loss were performed using the Kruskal–Wallis test followed by Dunn’s multiple comparisons test with Bonferroni correction. Safranin–O scoring data were also analysed using the Kruskal–Wallis test with Dunn’s post hoc multiple comparisons test. Due to the extremely limited sample size of the control group (*n* = 2), control samples were not included in inferential statistical comparisons and were interpreted descriptively. All statistical tests were two-tailed, and an alpha level of 0.05 was applied. Exact sample sizes were as follows: Group I, *n* = 5; Group II, *n* = 5; Group III, *n* = 4; controls, *n* = 2.

### ARRIVE compliance statement

All procedures involving animals were conducted in accordance with the ARRIVE guidelines (https://arriveguidelines.org). Experimental units, inclusion/exclusion criteria, study groups, outcome measures, and statistical analyses are reported in detail. All efforts were made to ensure transparency, reproducibility, and accurate reporting of all observations.

## Results

The study evaluated twelve dogs—six males and six females—and a total of fourteen stifle joints. Six stifles had medial patellar luxation and eight had lateral patellar luxation. Five stifles with (36%) Grade 2 patellar luxation (Group I), five (36%) with Grade 3 luxation (Group II), and four stifles (28%) with Grade 4 luxation (Group III) were consigned to different groups as per grade of luxation. The control group consisted of two stifle joints from dogs without patellar luxation or other orthopaedic abnormalities. Each group’s mean age and body weight distribution was statistically comparable. The median ages for Groups I, II, and III were 24, 11, and 18 months, respectively, whereas the overall age ranged from 5 to 42 months. Group I, Group II, and Group III had medians of 4.9, 13.3, and 8.3 kg, respectively, whereas the total body weight ranged from 3 to 40 kg. Half of the affected breeds were Pomeranians, followed by Labradors, Golden Retrievers, Cane Corso, Pugs, and Shih Tzus. All three groups showed similar levels of chronicity.

Articular cartilage sections from control dogs showed a normal cartilage surface and normal chondrocyte structure and distribution, with the surface exhibiting no signs of erosion and an intact superficial cartilage layer.

Group I (Grade 2 PL) cases showed minimal to mild erosions of the articular cartilage surface, along with mild changes in the superficial zone of cartilage surface. The chondrocytes showed a crinkled appearance and elongated nucleus, indicative of degenerative changes.

Group II (Grade 3 PL) articular cartilage sections showed mild to moderate damage of articular cartilage surface, indicated by fissure formations on the surface and minimal loss of chondrocytes as the predominant histopathological change. One section showed mild focal disruption of the cartilage surface, while another section exhibited a larger, irregular, and eroded surface. In a single specimen, a hypertrophic articular cartilage was observed merging with the subchondral bone.

**Fig. 1:**
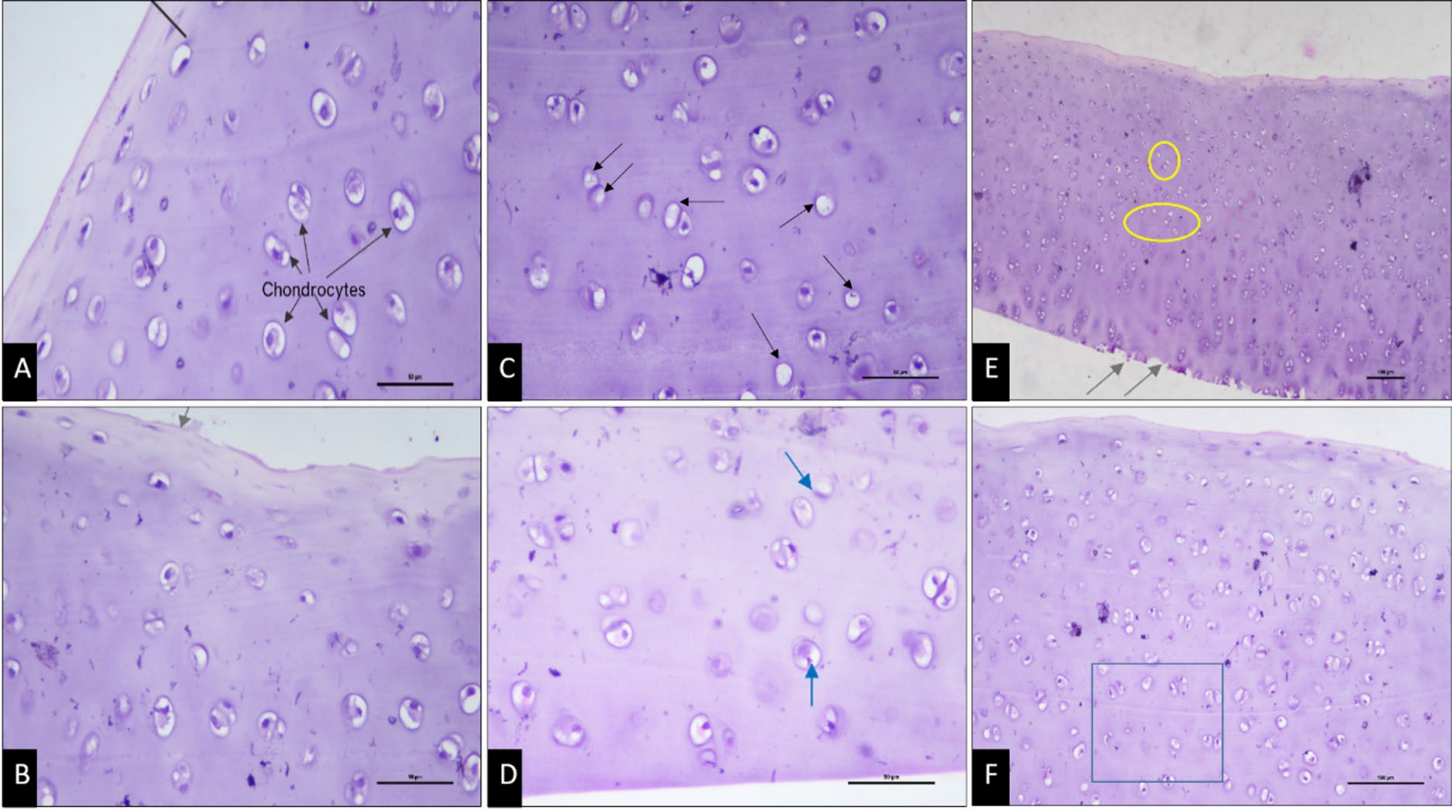
H&E-stained articular cartilage sections. **A** Normal cartilage surface and chondrocytes (Control group) – 40x, **B** Disruption of cartilage surface (arrow) (Group I; Grade 2 PL) – 40x, **C** Loss of chondrocytes (arrows) (Group II; Grade 3 PL) – 40x, **D** Disruptive changes in the chondrocytes (arrows) (Group III; Grade 4 PL) – 40x. **E** Representative overview image of section at 10x indicating Fibrillations at surface (arrows) and Loss of chondrocytes (circles) – 10x. **F** 20x overview image showing representative area of chondrocyte counting (square). All images represent representative sections from individual animals within each group (Control *n* = 2; Group I *n* = 5; Group II *n* = 5; Group III *n* = 4).

Group III (Grade 4 PL) cases showed marked structural disruptions in the chondrocytes, along with a relatively higher distribution of empty lacunae, suggestive of appreciable loss of chondrocytes. Fibrotic changes in the cartilage surface were observed in one specimen, amongst other degenerative changes in the structure of cartilage. Representative histological images are shown in Fig. 1.

### Primary outcome (chondrocyte loss)

**Fig. 2:**
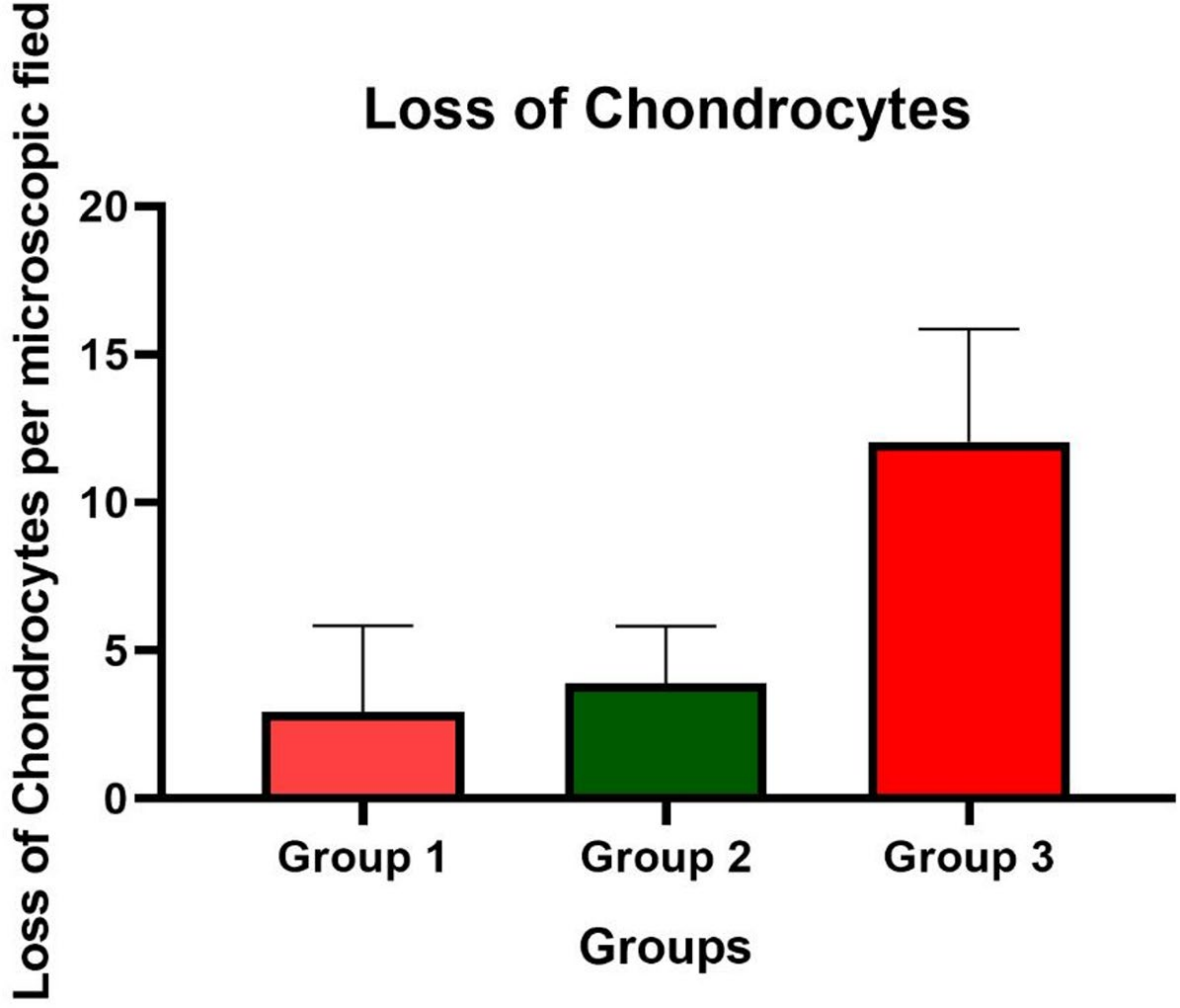
Chondrocyte loss per microscopic field across patellar luxation groups. Data are presented as mean ± SD. Statistical analysis was performed using the Kruskal–Wallis test (*p* < 0.0001).

Six microscopic fields per cartilage section were analysed from *n* = 5 cases in Groups I and II, *n* = 4 cases in Group III, and *n* = 2 control cases. Mean chondrocyte loss per microscopic field was 3.78 ± 4.71 (SD) in Group I, 3.89 ± 1.94 (SD) in Group II, and 12.06 ± 3.80 (SD) in Group III (Table 1). Normality testing demonstrated non-normal distribution of the data; therefore, non-parametric analysis was applied. Chondrocyte loss per microscopic field differed significantly among groups (Kruskal–Wallis test, H = 32.07, *p* < 0.0001). Post hoc pairwise comparisons using Dunn’s test with Bonferroni correction demonstrated significantly higher chondrocyte loss in Group III compared with Group I (p_adj = 3.47 × 10⁻⁶) and Group II (p_adj = 8.77 × 10⁻⁵), while no significant difference was observed between Group I and Group II (p_adj = 1.000). No chondrocyte loss was observed in control samples (*n* = 2), which were interpreted descriptively and were not included in inferential statistical testing due to the extremely limited sample size (Fig. 2).

**Table 1:** Descriptive statistics for chondrocyte loss per microscopic field.

Groups	Mean ± S.D. (empty lacunae/microscopic field)	Range (min.-max.)
Group I (*n* = 5)	3.78 ± 4.71	18 (0–18)
Group II (*n* = 5)	3.89 ± 1.94	6 (1–7)
Group III (*n* = 4)	12.06 ± 3.80	16 (4–20)

### Safranin – O staining

The Safranin-O fast green-stained sections of articular cartilage from the control dogs showed a normal safranin stain uptake in the superficial, intermediate, and territorial zones for both the orthopedically normal stifle joints’ cartilage samples. They were assigned a score of 9, indicative of normal proteoglycan content in the articular cartilage. Cartilage sections from patellar luxation groups showed reduced staining intensity with increasing grade, indicating decreased proteoglycan content.

**Fig. 3:**
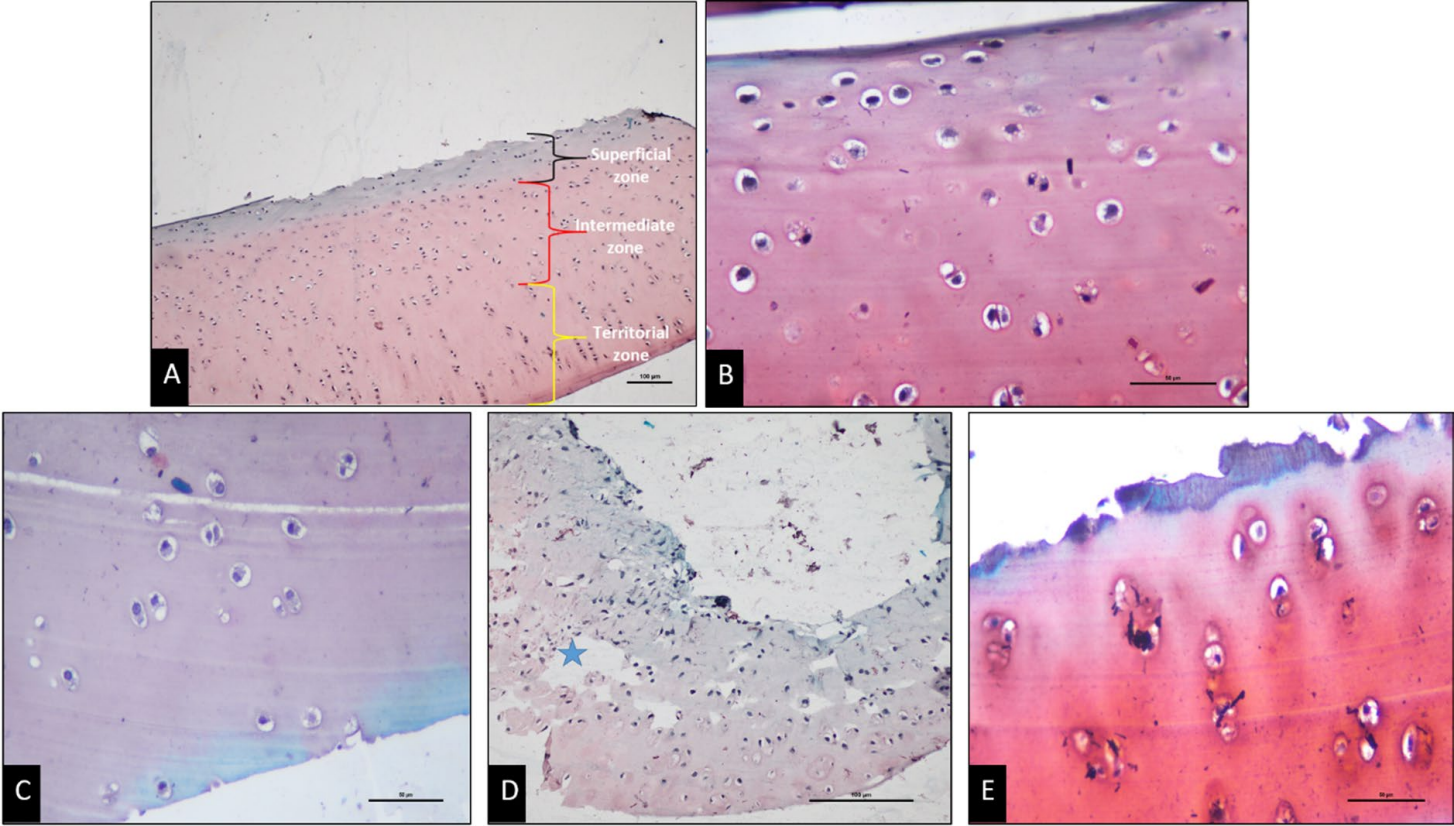
Safranin-O stained articular cartilage sections. **A** Overview representative image at 10x showing the zones used for Safranin-O scoring. **B** Normal Safranin-O staining with no loss of stain uptake (Control group) – 40x. **C** Mild loss of Safranin-O uptake in the superficial layer (Group I; Grade 2 luxation) – 40x. **D** Damaged cartilage (star) with complete loss of stain uptake in superficial zones (Group II; Grade 3 luxation) – 20x. **E** Damaged articular surface (arrow), with absent stain uptake in superficial zones and mild uptake in the intermediate zone (Group III; Grade 4 luxation) – 40x. Images represent representative sections from individual animals within each group (Control *n* = 2; Group I *n* = 5; Group II *n* = 5; Group III *n* = 4).

**Fig. 4:**
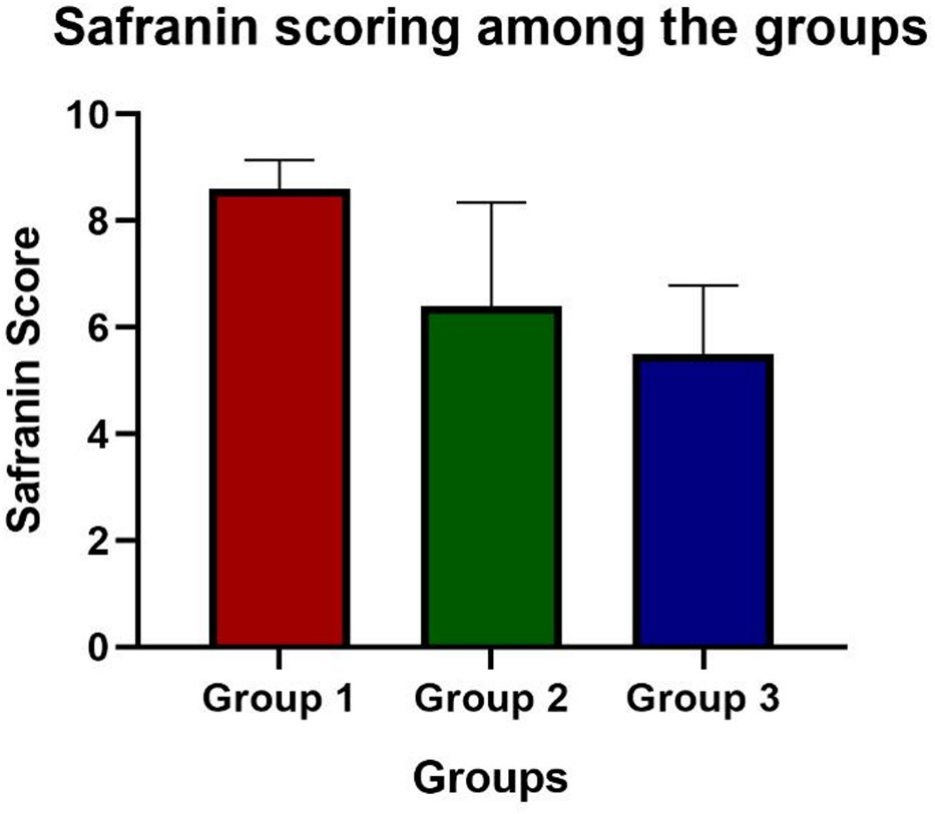
Safranin–O scoring of articular cartilage in patellar luxation groups. Data are presented as mean ± SD. Statistical analysis was performed using the Kruskal-Wallis test with Dunn’s post hoc test (*p* = 0.023).

Mean Safranin-O scores (± S.D.) were 8.6 ± 0.55 (range 8–9; *n* = 5) in Group I, 6.4 ± 1.95 (range 3–8; *n* = 5) in Group II, and 5.5 ± 1.29 (range 4–7; *n* = 4) in Group III. Statistical comparison among these groups using the Kruskal–Wallis test revealed a significant difference (H = 7.54, *p* = 0.023). Post hoc pairwise comparisons using Dunn’s test with Bonferroni correction showed trends toward lower scores in Groups II and III compared with Group I; however, these differences did not remain statistically significant after adjustment (*p* > 0.05). Control samples (*n* = 2) were interpreted descriptively and were not included in inferential statistical testing due to the extremely limited sample size. Representative histological sections are shown in Fig. 3, and groupwise statistical comparisons are illustrated in Fig. 4.

### Joint capsule

The joint capsule from control groups showed a normal synovial architecture with a one-cell-thick intimal lining layer and a synovial sub lining layer containing sparse lymphocytes, macrophages, and a dispersed distribution of fat cells, fibroblasts, and blood vessels.

In Group I (Grade 2 PL), joint capsule sections showed mild inflammatory changes including blood vessel proliferation, congestion (*n* = 3), mild haemorrhage (*n* = 1), and focal degenerative changes in one specimen.

In Group II (Grade 3 PL) cases, diffuse to widespread proliferation of fibroblast cells and lymphomononuclear cells infiltration was present at a local site, mild tendonitis with mononuclear cells and few fibroblasts around the blood vessels, accompanied by an increase in cellularity (*n* = 1); mild, scattered inflammatory changes, perivascular infiltration (*n* = 1), haemorrhage and extensive congestion (*n* = 1) and skeletal muscles and interspersed synovial membrane showing increased cellularity due to rise in fibroblasts and/or fibrocytes population were the altered features of joint capsule.

**Fig. 5:**
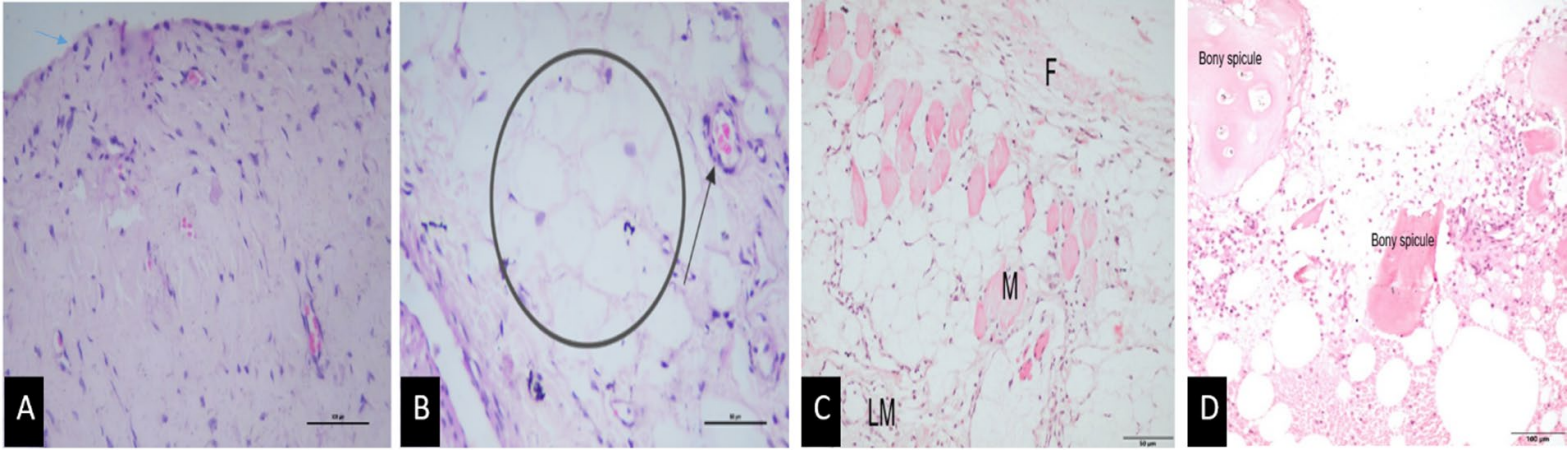
H&E-stained joint capsule. **A** Normal joint capsule with single-cell thick intimal lining layer (arrow) (Control group) – 20x. **B** Degeneration in joint capsule structure (circle) and congestion in blood vessels (arrow) (Group I; Grade 2 luxation) – 40x. **C** Mononuclear cells (M), fibroblast (F) infiltration around blood vessels and increased cellularity (Group II; Grade 3 luxation) – 40x. **D** Bony fragmentation and spicule formation with infiltration of inflammatory cells into the marrow component (Group III; Grade 4 luxation) – 20x. Images represent representative sections from individual animals within each group (Control *n* = 2; Group I *n* = 5; Group II *n* = 5; Group III *n* = 4).

In Group III (Grade 4 PL) cases, moderate to severe inflammatory changes were witnessed in the form of lympho-nuclear and polymorphonuclear (PMN) cells proliferation. Bony fragmentation with infiltration of inflammatory cells into the marrow component (*n* = 1) and poor bony osteoid matrix formation with deformed cartilage and loss of superficial cartilage layer, suggestive of osteophytosis was observed in one specimen. Representative images are shown in Fig. 5.

### Intra-operative findings

Gross intra-operative examination revealed osteophyte formations in seven out of 14 stifles (50%), of which there was mild osteophytosis in four cases and moderate to severe osteophytosis in three cases. The joint capsule was thickened in six cases (43%).

**Fig. 6:**
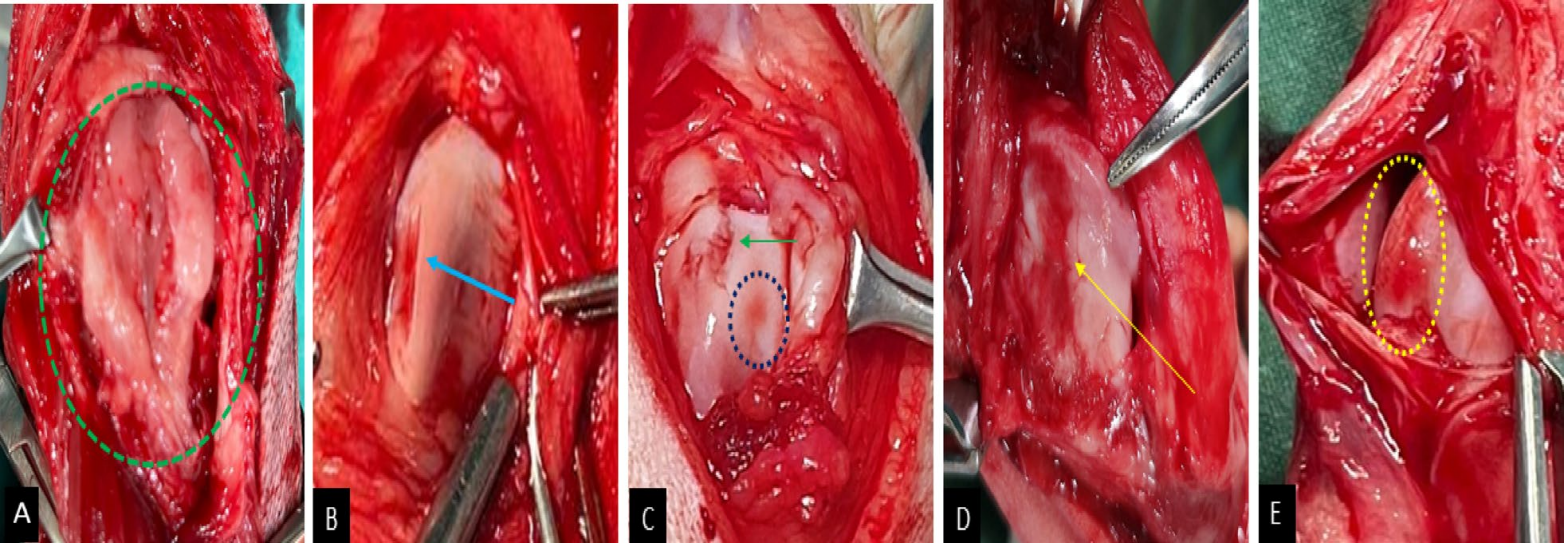
Gross, macroscopic alterations observed intra-operatively. **A** Osteophytosis and cartilage hypertrophy on femoral groove (green circle). **B** Articular cartilage erosive lesion at lateral femoral condyle (blue arrow). **C** Mild, focal erosive lesion at femoral groove (blue circle); neovascularization at medial femoral condyle (green arrow). **D** Articular cartilage erosion at femoral condyle and groove (yellow arrow). **E** Cartilage erosive lesion at medial femoral condyle (yellow circle).

Articular cartilage erosions were observed in eight of 14 dogs (57%). The frequency distribution of the articular cartilage erosive lesions showed that distal patellar and lateral trochlear surfaces had the highest prevalence of erosions in n = 4 (29%) cases. The proximal patella and femoral groove showed erosions in n = 3 (21%) stifles, while the medial trochlea surface and central patellar surfaces showed the lowest prevalence of articular cartilage erosions at 14% and 7% respectively. Representative gross findings are illustrated in Fig. 6.

## Discussion

The present study evaluated clinical, gross, and histopathological alterations of the stifle joint in dogs affected by patellar luxation, with a focus on articular cartilage and joint capsule changes across different grades of luxation. Histological examination demonstrated structural alterations of articular cartilage in patellar luxation–affected joints compared with control samples, including surface irregularities, altered chondrocyte morphology, increased numbers of empty lacunae, and reduced proteoglycan staining. Quantitative analysis demonstrated significantly greater chondrocyte loss in Grade 4 patellar luxation compared with lower grades, while no significant difference was observed between Grades 2 and 3. However, statistically significant differences among luxation grades were limited for proteoglycan content.

In the current study, histopathological alterations were most consistently observed in the superficial zones of articular cartilage, with features such as surface fibrillation, altered chondrocyte morphology, and empty lacunae. These findings are in agreement with earlier reports describing superficial zone involvement as a predominant feature of cartilage degeneration in medial patellar luxation–affected stifles^12^. Similar to the observations of Wander et al., Safranin-O staining in the present study did not demonstrate statistically significant differences among luxation grades, although staining intensity was significantly reduced in Group III (Grade 4 PL) compared with control samples, suggesting trends toward proteoglycan loss rather than definitive progression.

Joint capsule changes observed in the present study, including fibroblast proliferation, increased cellularity, and inflammatory cell infiltration, are consistent with previously reported synovial and capsular alterations in dogs with medial patellar luxation^13^. These findings support the concept that patellar luxation is associated not only with cartilage changes but also with reactive alterations of periarticular soft tissues, which may contribute to joint dysfunction and disease progression.

Gross intra-operative findings in this study revealed articular cartilage erosions in 57% of affected stifles, with the distal patella and lateral trochlear surfaces being the most frequently involved sites. This prevalence is comparable to earlier studies reporting cartilage erosions in 39–64% of medial patellar luxation cases^3,4^. Previous reports have identified patellar articular surfaces and medial femoral trochlear ridges as the most common sites of erosion^1,5^, findings that are broadly consistent with the lesion distribution observed in the present study.

Although increased chondrocyte loss was observed in higher-grade luxation, the limited number of statistically significant differences across grades, particularly for proteoglycan content, indicates that these findings should be interpreted as associations rather than definitive evidence of progressive degeneration. Furthermore, as the data were obtained at a single time point, the present findings cannot determine whether the observed histological alterations represent progressive changes over time or are directly driven by increasing luxation severity.

The objective of the present study was to evaluate structural alterations of articular cartilage and joint capsule across different grades of patellar luxation and to correlate these findings with gross intra-operative observations. The results demonstrate that although statistically significant differences among luxation grades were limited, higher-grade luxation was consistently associated with greater chondrocyte loss, more pronounced structural disruption of cartilage, and reduced Safranin-O staining, indicating diminished proteoglycan content. These findings indicate an association between higher grades of patellar luxation and a greater extent of cartilage degeneration, although statistical separation between intermediate grades was limited in this small clinical dataset. From a clinical perspective, the presence of histological cartilage degeneration in moderate and severe grades highlights the importance of timely surgical correction and careful postoperative management to minimize further joint degeneration and the development of secondary osteoarthritis.

Overall, the findings of the present study support existing evidence that patellar luxation is associated with structural and biochemical alterations of both articular cartilage and joint capsule, while also highlighting variability in the extent and distribution of these changes. Future studies incorporating larger sample sizes, longitudinal follow-up, and standardized histological scoring systems may further clarify the relationship between luxation severity and joint degeneration.

### Limitations of the study

This study has several limitations that should be considered when interpreting the findings. First, the sample size was limited, particularly in Group III (*n* = 4) and the control group (*n* = 2), which may have reduced statistical power and limited detection of smaller group differences. Second, although normality testing demonstrated non-normal distribution of chondrocyte loss data and appropriate non-parametric statistical methods were applied, the small sample size limits generalizability of the results. Third, the study design did not incorporate randomisation or blinding, as all eligible clinical cases were included and histopathological evaluation was performed by the same clinical team, which may introduce observer bias. Additionally, the cross-sectional design precludes assessment of temporal progression or causal relationships between patellar luxation severity and histological changes. Finally, the considerable variation in body size and breed composition among the dogs may have influenced joint loading patterns and cartilage response, contributing to inter-individual variability. Future studies employing larger, more uniform cohorts, blinded assessments, and longitudinal designs are warranted to strengthen causal inference and improve external validity.

## Conclusion

This study demonstrates that dogs affected by patellar luxation exhibit histopathological and gross alterations of the stifle joint, including degenerative and inflammatory changes in both articular cartilage and joint capsule. Although statistically significant differences among patellar luxation grades were limited, higher-grade cases were consistently associated with greater chondrocyte loss, structural disruption of cartilage, and reduced Safranin–O staining compared with control joints, indicating compromise of the structural and biochemical integrity of stifle joint tissues. These findings support an association between increasing severity of patellar luxation and joint tissue degeneration and underscore the clinical importance of early diagnosis and appropriate surgical management to minimise secondary osteoarthritic changes. Larger studies with longitudinal follow-up are warranted to further clarify the relationship between luxation severity and the progression of joint pathology.

## Data Availability

The datasets generated and analysed during the current study are available from the corresponding author on reasonable request.
